# Dual antibacterial mechanism of [K4K15]CZS-1 against *Salmonella* Typhimurium: a membrane active and intracellular-targeting antimicrobial peptide

**DOI:** 10.3389/fmicb.2023.1320154

**Published:** 2023-12-14

**Authors:** Sebastián Bermúdez-Puga, Meriellen Dias, Taciana Freire de Oliveira, Carlos Miguel Nóbrega Mendonça, Sonia Regina Yokomizo de Almeida, Enrique Eduardo Rozas, Claudio Augusto Oller do Nascimento, Maria Anita Mendes, Pamela Oliveira De Souza de Azevedo, José R. Almeida, Carolina Proaño-Bolaños, Ricardo Pinheiro de Souza Oliveira

**Affiliations:** ^1^Microbial Biomolecules Laboratory, Faculty of Pharmaceutical Sciences, University of São Paulo, São Paulo, Brazil; ^2^Department of Anatomy, Biomedical Sciences Institute, University of São Paulo, São Paulo, Brazil; ^3^Dempster MS Lab, Chemical Engineering Department of Polytechnic School of University of São Paulo, São Paulo, Brazil; ^4^Biomolecules Discovery Group, Universidad Regional Amazónica Ikiam, Tena, Napo, Ecuador; ^5^School of Pharmacy, University of Reading, Reading, United Kingdom

**Keywords:** antimicrobial peptides, cruzioseptin, mechanism of action, metabolomics, membranolytic effect

## Abstract

*Salmonella* genus is a leading cause of food-borne infections with strong public health impact and economic ramifications. The development of antimicrobial resistance added complexity to this scenario and turned the antibiotic drug discovery into a highly important challenge. The screening of peptides has served as a successful discovery platform to design new antibiotic candidates. Motivated by this, the antimicrobial and cytotoxic properties of three cruzioseptins against *Salmonella* Typhimurium and RAW 264.7 murine macrophage cells, respectively, were investigated. [K4K15]CZS-1 was the most potent antimicrobial peptide identified in the screening step with a minimum inhibitory concentration (MIC) of 16 μg/mL (7.26 μM) and moderate cytotoxicity. From a structural point of view, *in vitro* and *in silico* techniques evidenced that [K4K15]CZS-1 is a α-helical cationic antimicrobial peptide. In order to capture mechanistic details and fully decipher their antibacterial action, we adopted a multidimensional approach, including spectroscopy, electron microscopy and omics analysis. In general lines, [K4K15]CZS-1 caused membrane damage, intracellular alterations in *Salmonella* and modulated metabolic pathways, such as the tricarboxylic acid (TCA) cycle, fatty acid biosynthesis, and lipid metabolism. Overall, these findings provide deeper insights into the antibacterial properties and multidimensional mode of action of [K4K15]CZS-1 against *Salmonella* Typhimurium. In summary, this study represents a first step toward the screening of membrane-acting and intracellular-targeting peptides as potential bio-preservatives to prevent foodborne outbreaks caused by *Salmonella*.

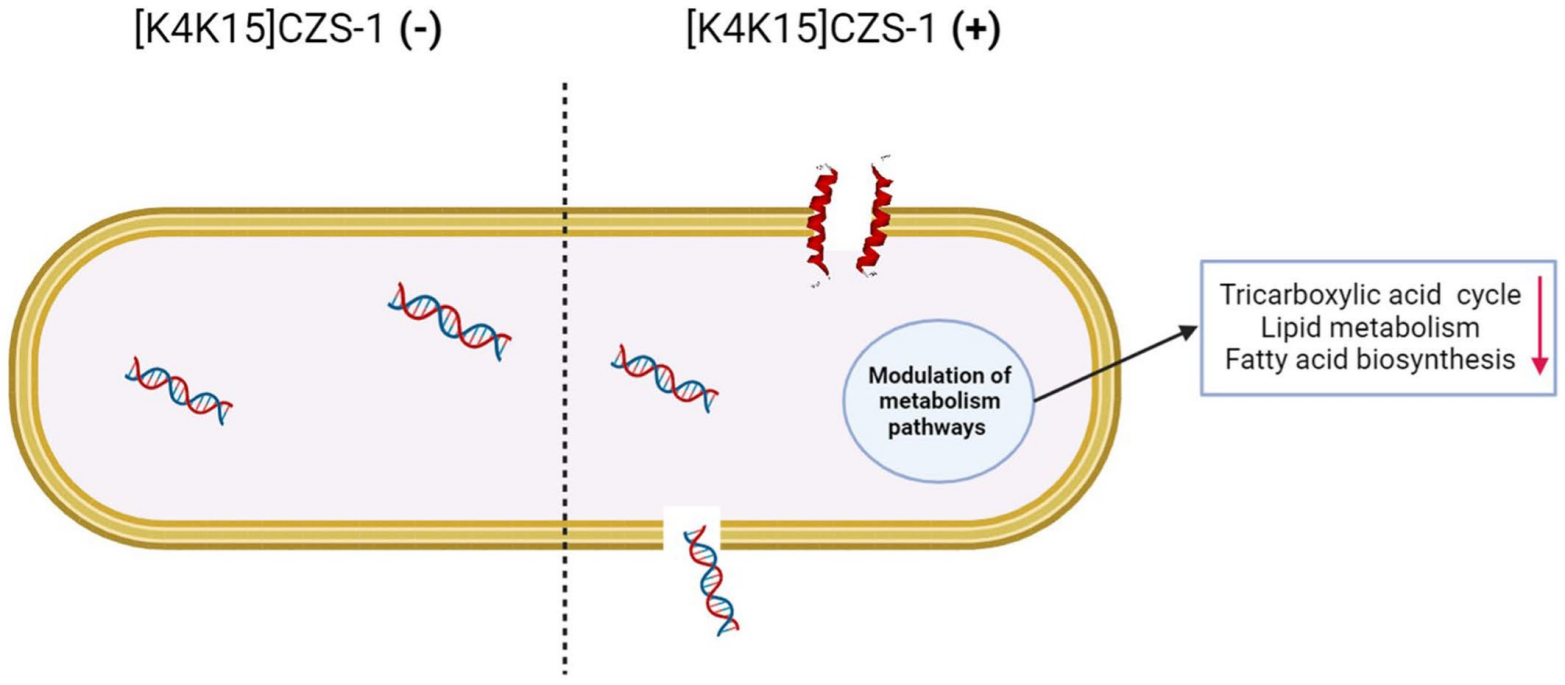

## 1 Introduction

Foodborne diseases constitute a major problem due to the millions of infections and thousands of deaths generated every year. *Salmonella* spp., *Campylobacter* spp., and *Listeria* spp. are some of the bacterial pathogens involved in this global threat to food safety and human health (Gutiérrez-Del-Río et al., 2018). Several studies have pointed out that *Salmonella enterica* subsp. *enterica* serovar Typhimurium (*S.* Typhimurium) is the most common etiological agent of food-poisoning outbreaks caused by this genus (Gordon et al., 2008; Xia et al., 2015). *Salmonella* contamination can drastically affect the production chain and survive for extended periods in low-moisture conditions due to simple nutritional requirements (Sánchez-Maldonado et al., 2018; Finger et al., 2019; Ehuwa et al., 2021).

To date, traditional antibiotics and antibacterial physical treatments have been widely used to inhibit microbial growth and increase the shelf-life of food (Rai et al., 2016; Adaro et al., 2023). However, the prolonged use of antibiotics have been associated with the emergence of multi-resistant microorganisms, representing a serious public and life-threatening health problem (Ehuwa et al., 2021; Moretta et al., 2021). As a result, a roadmap to tackle this challenge must include the discovery of antimicrobial chemical scaffolds that are less susceptible to bacterial resistance and compatible with safety and quality of food.

Antimicrobial peptides (AMPs) have been becoming attractive lead compounds in ambitious programs to produce the next-generation of antibiotics for application in the food industry (Lima et al., 2021). AMPs combine several better properties compared to conventional chemical preservatives, such as high antimicrobial potency, low propensity to develop bacterial resistance, low toxicity, broad-spectrum of antimicrobial activity, and multiple mode of action (Benfield and Henriques, 2020; Li et al., 2021). An example of peptides candidates are cruzioseptins, a frog AMP family isolated from *Cruziohyla calcarifer* (Proaño-Bolaños et al., 2016). These multifunctional short molecules have shown antibacterial, antifungal, and antiprotozoal properties (Proaño-Bolaños et al., 2016; Mendes et al., 2020; Cuesta et al., 2021). Their sequences have been employed as a template to generate more active and selective cruzioseptin analogs. Fine-tuning cruzioseptins led to the identification of a potent antibacterial peptide named [K4K15]CZS-1 with reduced toxicity (Bermúdez-Puga et al., 2023). The antimicrobial mechanism of natural and engineered cruzioseptins on *Staphylococcus aureus, Escherichia coli*, and *Leishmania* spp. seems to involve the electrostatic interaction and consequently disrupting of cell membrane (Mendes et al., 2020; Valdivieso-Rivera et al., 2022; Bermúdez-Puga et al., 2023).

Many studies have focused on the membranolytic action of peptides on pathogens associated nosocomial hospital-acquired infections. A limited number of studies have explored the effect of peptide candidates on bacteria causing food poisoning and their mode of action from a multidimensional perspective. It is crucial to clarify in depth how AMPs works to avoid the resistance in microbial pathogens and bring them from screening step to the point of therapeutic use (Benfield and Henriques, 2020; Li et al., 2021). Traditionally, electron microscopy have been widely employed to visualize the membrane-damaging activities of peptides. In line with this, modern technologies, such as the metabolomics constitute valuable analytical tools to build a comprehensive understanding of biochemical alterations in pathogens after AMP exposure. However, there are few examples of integrative efforts combining different strategies to explore this mechanistic scenario (Bo et al., 2014; Sun et al., 2023). Based on this, the aim of this work was to expand the current understanding of the potential applications of CZS-9, CZS12, [K4K15]CZS-1, by examining the activities against *S.* Typhimurium and RAW 264.7 murine macrophage cells. In addition, the mechanisms underlying the antimicrobial properties were investigated integrating electron microscopy and metabolomics approaches.

## 2 Materials and methods

### 2.1 Solid-phase peptide synthesis

Three peptides CZS-9: GFLDVITHVGKAVGKAALNAVNEMVNQ-NH_2_, CZS-12: GFLDVVKHVGKAVGKAALNAVNDLVNQ-NH_2_, and [K4K15]CZS-1: GFLKIVKGVGKVALKAVSKLF-NH_2_ were synthesized by solid-phase peptide synthesis using the Fmoc chemistry in a Liberty Blue automated microwave peptide synthesizer (CEM Corporation). Fmoc Rink Amide (LL) (CEM Corporation, USA) was used as the solid resin support, N, N′-dimethylformamide was utilized as the main solvent for the synthesis, and diisopropylcarbodiimide/Oxyma base was employed as an activator of amino acids during the coupling process. Fmoc protector groups were removed with piperidine 20%. While an acidic cocktail containing trifluoroacetic acid (TFA) 92.5%, triisopropyl silane 2.5%, 3,6-dioxa-1,8-octanedithiol 2.5%, and water 2.5%, was used to cleave the peptide product from the resin and to remove the protector groups from the side chains of amino acids. Crude peptides were washed and precipitated with cold diethyl ether. Finally, the synthesized peptides were freeze-dried in vacuum conditions at −80°C at a pressure of 0.09 mT and stored at −20°C.

### 2.2 Characterization and purification of synthetic peptides

The purity of crude peptides was assessed by reverse-phase high performance liquid chromatography (RP-HPLC) equipped with a two-pump chromatograph (Agilent Technologies) coupled to a C18 analytical column (250 × 4.6 mm, 5 μm) and a UV–VIS detector at 220 nm. In brief, 1 mg of peptide was dissolved in 200 μL of water/TFA solution (99.9/0.1 v/v). Peptides were eluted by applying a gradient linear system from 99% mobile phase A (99.9% water/0.1% TFA) to 99% mobile phase B (99.9% acetonitrile/0.1% TFA) at a flow rate of 1 mL/min for 55 min. Additionally, the molecular mass of peptides was corroborated by matrix-assisted laser ionization/desorption time-of-flight mass spectrometry (MALDI-TOF MS) (Bruker Daltonics).

Crude peptides that did not present a purity >80% were purified by RP-HPLC using a two-pump chromatograph (Agilent technologies) coupled to a C18 analytical column (50 × 21.2 mm, 5 μm) and a UV–VIS detector at 220 nm. In summary, 15 mg of peptides were dissolved in 800 μL water/TFA solution (99.9/0.1 v/v) and filtered through a 0.22 μm membrane filter. The sample was injected at a flow rate of 2.5 mL/min for 10 min with a linear gradient from 53% mobile phase A to 53% mobile phase B. The purified peptide was lyophilized, and the analysis of purity was evaluated using the methodology mentioned above.

### 2.3 Determination of secondary structures of AMPs by circular dichroism (CD)

The secondary structure of AMPs in different environments was determined on a Jasco J-720 spectropolarimeter (Jasco, Tokyo, Japan) at 25°C using a 2 mm path-length rectangular quartz cell and baseline corrected using proper control. For this assay, peptides, with a purity of >90%, were dissolved in deionized water at pH 7 (mimicking the aqueous environment) or 30 mM SDS micelles (mimicking a prokaryotic cell membrane with a negative charge) at final concentration of 0.1 mg/mL (CZS-9: 0.42 mM, CZS-12: 0.38 mM, [K4K15]CZS-1: 0.45 mM). The spectra were recorded between 190 and 260 nm using the following parameters: 20 nm/min scan speed, 0.2 nm data pitch, 1 s response time, 1 nm band width, and 3 accumulations. The acquired circular dichroism (CD) signal spectra were then converted to mean residue ellipticity with the following equation:


$$\theta_{M}=\frac{\theta_{\mathrm{abs}}}{c*l*n}$$

where $\theta_{M}$ is the mean residue ellipticity (deg*cm^2^*dmol^−1^), θ_abs_ is the observed ellipticity corrected for the buffer at a given wavelength (mdeg), c is the peptide concentration (mM), l is the path length (mm), and n is the number of amino acids.

### 2.4 Prediction of physicochemical properties and three-dimensional structures of peptides

The HELIQUEST server was used to predict the physicochemical properties and the helical wheel diagram of peptides (Gautier et al., 2008). The theoretical mass was calculated by the Bachem Peptide Calculator.^1^ In addition, the three-dimensional structure of peptides was obtained using the I-TASSER server (Roy et al., 2010) and the models were visualized with BIOVIA Discovery Studio v.4.5 (BIOVIA, San Diego, CA, USA).

### 2.5 Minimum inhibitory concentration (MIC) and minimum bactericidal concentration (MBC) assays

The MIC and MBC of AMPs was determined against *Salmonella enterica* subsp. *enterica* serovar Typhimurium, following the protocol by Proaño-Bolaños et al. (2016) with minor modifications. In summary, different peptide concentrations (1–512 μg/mL) were prepared by serial dilution with dimethyl sulfoxide (DMSO). Pathogenic bacterium was grown in Brain Heart Infusion (BHI) medium at 37°C until to reach the concentration of 1 × 10^6^ UFC/mL. Subsequently, the bacterial suspension was mixed with the peptide dilutions and were transferred to a 96 well sterile microplate. A DMSO solution instead of peptide and BHI medium without bacteria were included as controls. The microplates were incubated at 37°C for 18 h. After the incubation time, the absorbance was measured at 600 nm using a microplate reader (Synergy HTX, Biotek). The MIC value was determined as the minimal concentration that did not show bacterial growth in the measurement by optical density. Finally, 10 μL of each concentration without bacterial growth was subcultured on BHI agar (1% v/v) plates and incubated at 37°C overnight. MBC value was defined as the lowest peptide concentration without any bacterial growth occurrence.

### 2.6 Cell-based assay for assessing the peptide toxicity

RAW-264.7 macrophage cell line was grown in Dulbecco’s Modified Eagle Medium – high glucose (DMEM) (Thermo Fisher Scientific) supplemented with 10% fetal bovine serum (FBS) (Thermo Fischer Scientific) and 1% antibiotic-antimycotic solution (Thermo Fisher Scientific) and then incubated at 37°C under humidified conditions in a 5% CO_2_ atmosphere after cells reached 70–80% confluency. The growth medium was refreshed every 2–3 days. The cells were washed twice with phosphate buffer saline (PBS) and harvested using 0.25% trypsin–EDTA (Sigma-Aldrich Corporation, St. Louis, MO, USA).

For cytotoxicity evaluation, cells were plated in 96-well plates (100 μL/well) at a density of 2×10^4^ cells/mL and incubated for 24 h at 37°C in 5% CO_2_. Subsequently, the medium was removed from the plate, and the adherent cells were incubated with 90 μL of cell culture media and 10 μL of pure peptide (1 μg/mL to 512 μg/mL). The plates were incubated for 24 h at 37°C in 5% CO_2_, and after incubation, 100 μL of 3-(4,5-dimethylthiazol-2- yl)-2,5-diphenyl tetrazolium bromide (MTT) solution (5 mg/mL) (Sigma-Aldrich Corporation, St. Louis, MO, USA) were added to each well. The plates were further incubated at 37°C in 5% CO_2_ for 3 h. Afterwards, the medium was removed and formazan crystals were dissolved with 100 μL of DMSO. The absorbance was measured at 540 nm and the percentages of viable cells were calculated:


$$\%\mathrm{Cell}\;\mathrm{viability}=\frac{\mathrm{Mean}\;\mathrm{Optical}\;\mathrm{density}\;\mathrm{treated}}{\mathrm{Mean}\;\mathrm{Optical}\;\mathrm{density}\;\mathrm{untreated}}x\;100$$

Cells treated with Triton x-100 (0.1% v/v) and DMEM medium with 1% DMSO (v/v) were used as positive and negative controls, respectively. Five replicates were made for each AMPs sample.

### 2.7 Measurement of release nucleic acids

To evaluate if the bacterial membrane is compromised by the [K4K15]CZS-1 effect, a nucleic acid release assay was employed according to Tang et al. (2008) with minor modifications. Briefly, *Salmonella* Typhimurium was growing exponentially on BHI at 37°C until reaching an optical density (OD) of 0.4. The bacterial suspension (1 × 10^8^ UFC/mL) was washed twice and resuspended with PBS. The pathogenic bacteria were incubated at 37°C for 2 h with the peptide at a final concentration of 1 xMIC, 2 xMIC, and 4 xMIC. As a negative control, bacteria were incubated with PBS 1X. Subsequently, samples were filtered using 0.22 μm syringe filters to remove the bacterial cells. Finally, the supernatant was measured at 260 nm using a microplate reader (Synergy HTX, Biotek).

### 2.8 Scanning electron microscopy

The morphological changes of the bacterial membrane after treatment with [K4K15]CZS-1 were observed under scanning electron microscopy (SEM). *Salmonella* Typhimurium was grown in BHI at 37°C to mid-logarithmic phase. Later, the bacterial pellet was harvested by centrifugation at 4000 rpm for 10 min, washed with PBS 1X for two times, and resuspended in PBS 1X. Bacterial cells were incubated at 37°C for 2 h with 4 xMIC of [K4K15]CZS-1. Subsequently, cells were fixed with 2.5% (v/v) glutaraldehyde at 4°C overnight. The bacteria cells were washed twice with PBS to remove the glutaraldehyde and dehydrated with a graded ethanol series (50, 70, 90, and 100% ethanol) at each dilution for 10 min. The same procedure was repeated for the control, however, bacterial cells were not incubated with the peptide. Finally, all the samples were transferred to a coverslip, left to dry at room temperature for 1 h, and coated 10 s with gold. It was visualized under a scanning electron microscope with 15 kV resolution (LEO 435VP).

### 2.9 Transmission electron microscopy

The intracellular alterations and changes in structural integrity of *S.* Typhimurium were investigated under transmission electron microscopy (TEM). In summary, the pretreatment of bacterial cells was under the same conditions described for the SEM assay. After pre-fixation with 2.5% glutaraldehyde, the bacterial pellet was washed twice with PBS and subsequently post-fixed with 2% osmium tetroxide in PBS for 2 h. The bacterial cells were washed twice with PBS and dehydrated in a graded ethanol series (50, 70, 90, and 100% for 8 min in each step) and incubated in 100% ethanol (10 min), a 1:1 mixture of 100% ethanol and acetone (10 min), and pure acetone (10 min). The samples were immersed in a mixture of absolute acetone and epoxy resin (1:1) for 30 min and pure epoxy resin overnight. Finally, an ultramicrotome was used to cut ultrathin slices, which were then stained with uranyl acetate and lead citrate. Samples were examined using a transmission electron microscope (Morgagni 268D).

### 2.10 Metabolomics analysis of *Salmonella* Typhimurium treated with [K4K15]CZS-1

Perturbations in the intracellular metabolite profiling of bacterial cells induced by [K4K15]CZS-1 was analyzed by gas chromatography coupled with a mass spectrometer (GC–MS). To this end, *S.* Typhimurium cells were grown in the presence (0.5 and 1 xMIC) or absence of the peptide [K4K15]CZS-1 for 18 h. After the incubation period, the bacterial pellet was harvested by centrifugation at 4000 rpm for 10 min and then washed twice with PBS 1X. Later, the bacterial pellet was dried in a vacuum concentrator. The intracellular metabolites were extracted using 1 mL of methanol, followed by a shake for 30 s, a sonication process for 15 min, and finally drying in a vacuum centrifuge. Three biological replicates were performed for each sample.

The derivatization process was carried out by adding 100 μL of a solution with proportions of N-methyl-N-(trimethylsilyl) trifluoroacetamide (50%) and a solvent mixture: acetonitrile/dichloromethane/cyclohexane (5:4:1) and 5% trimethylamine (50%). The samples were agitated for 30 s and were placed in a 60°C thermal bath for 1 h. Following that, the samples were centrifuged for 2 min at 12,000 g. GC–MS was used to analyze the supernatant.

### 2.11 Secretome analysis of *Salmonella* Typhimurium treated with [K4K15]CZS-1

Alterations in the extracellular metabolite profiling of *S.* Typhimurium incubated with or without [K4K15]CZS-1 were studied by GC–MS. In this assay, the cell-free supernatant (CFS) of *S.* Typhimurium incubated with (0.5 and 1 xMIC of [K4K15]CZS-1 for 18 h) or without peptides was dried in a vacuum concentrator. Later, extracellular metabolites present in the CFS were obtained by adding 300 μL of methanol and agitating it for 2 min. The sample was then dried in the vacuum centrifuge. Three biological replicates were performed for each sample. Finally, the derivatization was performed according to the methodology described previously, and the metabolites were analyzed using GC–MS.

### 2.12 Identification of metabolites by GC–MS

Analysis was performed by a GS-MS system (Shimadzu TQ-8050). Two μL of sample was injected with a split ratio of 1:10. Helium was used as a carrier gas at a constant flow rate of 1 mL/min. The GCMSsolution Smart MRM version 4.2. GC–MS analysis was utilized, and the running time of each sample was 67 min. The injection, interface, and ionization source temperatures were 280°C, 280°C, and 250°C, respectively. The initial temperature was set to 100°C, then raised to 320°C in a linear ramp of 4°C/min, and finally maintained at 320°C for 8 min. The mass fingerprints of 568 compounds are included in the Shimadzu Smart Metabolites Database: metabolites identified if their scanned ion pairs are 80% compatible with the database.

### 2.13 Data analysis

#### 2.13.1 Omics analysis

The GC–MS/MS data were analyzed using Web-based MetaboAnalyst 5.0 free software (updated and maintained by the Xia Lab at McGill University). The metabolite relative abundances were submitted to multivariate statistical analyses, such as principal component analysis (PCA) and partial least squares discriminant analysis (PLS-DA), which were performed with MetaboAnalyst 5.0 free software. Additionally, differentially expressed metabolites were detected by a student’s t-test with a *p* value of less than 0.05 and the main metabolites were shown using a hierarchical cluster graph. The determination of stress indicators (Biomarker meta-analysis) was carried out using the meta-analysis approach based on the combination of the *p*-values. The identified metabolites, with mean local confidence scores ≥80%, were compared in the KEGG PATHWAY database.

#### 2.13.2 Statistical analysis

GraphPad Prism 8.0 software (GraphPad Software Inc., San Diego, CA, USA) was used for statistical analysis. The acquired data were reported as mean ± standard deviation and evaluated using analysis of variance (ANOVA), followed by post-hoc Tukey multiple comparison. *p* < 0.05 was considered as significant.

## 3 Results

### 3.1 Sequence analysis and physicochemical properties of AMPs

Two cruzioseptins, CZS-9 and CZS-12, isolated from the skin secretion of *Cruziohyla calcarifer*, and a cruzioseptin analog, [K4K15]CZS-1, were selected for this study (Table 1). Natural cruzioseptins present a high degree of identity in the primary structure, and both peptides have a length of 27 amino acids. However, the net charge varies on CZS-9 and CZS-12, with values of +1 and + 2, respectively. On the other hand, [K4K15]CZS-1 has a net charge of +6, a length of 21 amino acids, and higher hydrophobicity than other cruzioseptins.

**Table 1:** Primary sequences of cruzioseptins and their predicted physicochemical properties.

AMPs	Sequence	Theoretical mass (Da)	Hydrofobicity	Net charge	Length (aa)	Reference
CZS-9	GFLDVITHVGKAVGKAALNAVNEMVNQ-NH_2_	2795.26	0.393	1	27	Proaño-Bolaños et al. (2016)
CZS-12	GFLDVVKHVGKAVGKAALNAVNDLVNQ-NH_2_	2776.22	0.335	2	27	Proaño-Bolaños et al. (2016)
[K4K15]CZS-1	GFLKIVKGVGKVALKAVSKLF-NH_2_	2201.8	0.523	6	21	Bermúdez-Puga et al. (2023)

### 3.2 Synthesis, characterization, and purification of AMPs

The synthetic AMPs presented a purity of 82.37% for CZS-9, 89.92% for CZS-12, and 64.48% for [K4K15]CZS-1 (Supplementary Figure S1). Subsequently, a purification step of [K4K15]CZS-1 was performed and its analysis by RP-HPLC indicated a 94.48% purity. Additionally, the expected molecular weight of these three peptides is in accordance with the theoretical mass, confirming their chemical identities (Supplementary Figure S1; Table 1).

### 3.3 Secondary structures and *in silico* three dimensional structures

**Figure 1:**
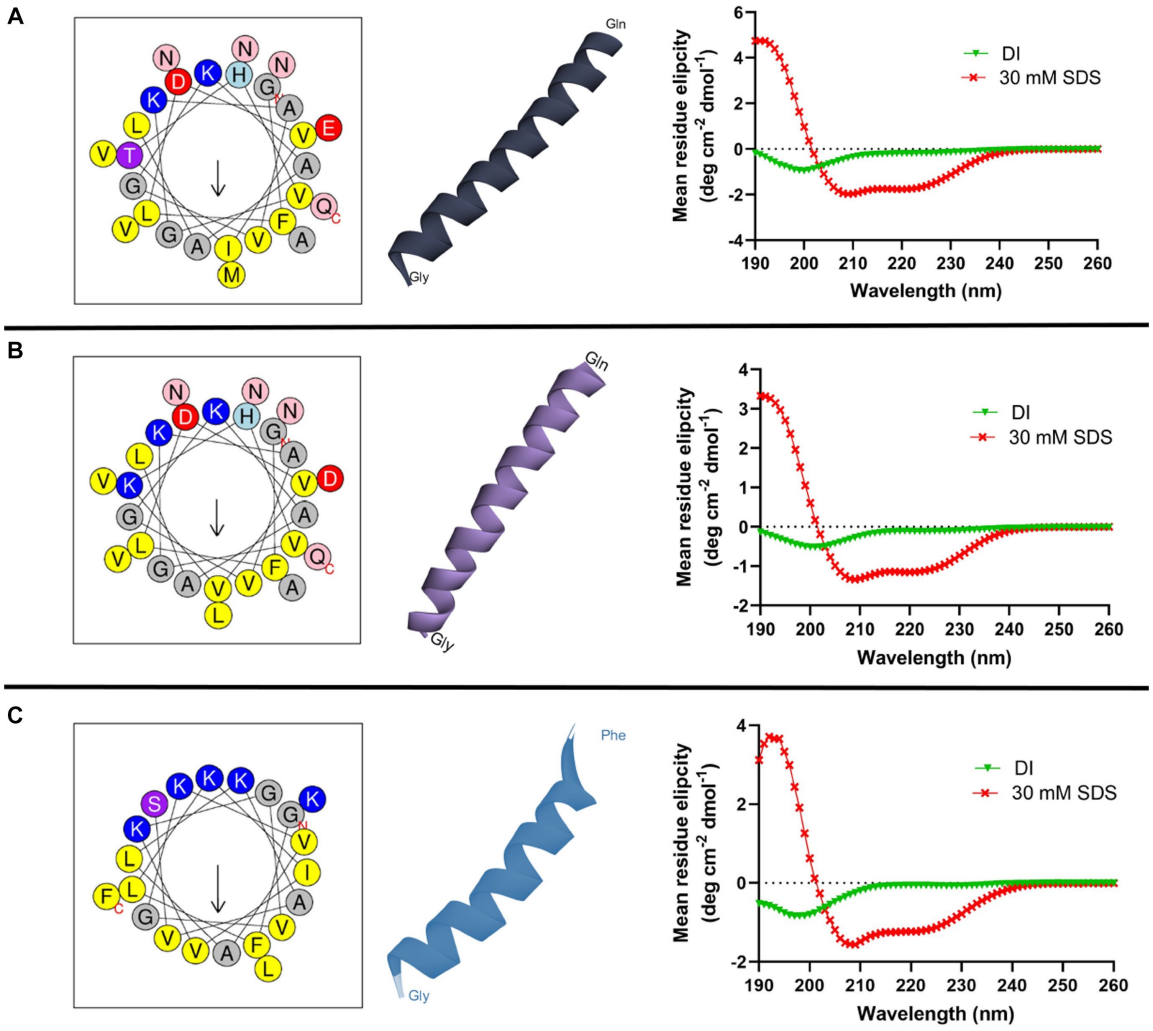
Helical wheel diagram, three-dimensional structure, and circular dichroism spectra of cruzioseptins: **(A)** CZS-9, **(B)** CZS-12, and **(C)** [K4K15]CZS-1. In the helical wheel plot, non-polar residues are shown in yellow, polar residues in purple, basic residues in blue/blue light, and acid residues in red. Uncharged residues such as alanine and glycine are in gray, while asparagine and glutamine are in pink. The secondary structure of peptides was investigated in deionized water (DI) and 30 mM SDS micelles. All peptides showed a random coil structure and a α-helix structure in an aqueous solution and an SDS solution, respectively.

*In silico* tools were used to obtain the helical wheel projection and the three-dimensional structures of all AMPs (Figure 1). According to HELIQUEST and I-TASSER analysis, all three peptides tended to form an α-helix with an amphipathic structure. In addition, the secondary structure of AMPs in the presence of aqueous solution or 30 mM SDS micelles was investigated by CD spectroscopy (Figure 1). All peptides display a random coil structure in aqueous solution, as indicated by the appearance of a negative peak near at 200 nm. On the other hand, all peptides form an α-helix in the SDS micelles, which is determined by the two negative bands at 208 and 222 nm and one positive band at 192 nm.

### 3.4 Antibacterial and cytotoxic activity of cruzioseptins

The antibacterial effect of the three peptides against *S.* Typhimurium was evaluated and summarized in Table 2. CZS-9 has no antibacterial activity against *S.* Typhimurium. CZS-12 was only active at the highest concentration tested. Whereas, the engineered peptide, [K4K15]CZS-1, was highly active with a MIC value of 7.26 μM. On the other hand, the MBC value of [K4K15]CZS-1 showed a 2 fold increase in comparison with the MIC values.

**Table 2:** Minimum inhibitory concentration (MIC) and minimum bactericidal concentration (MBC) of cruzioseptin family members.

Strain	MIC μg/mL (μM)		
	CZS-9	CZS-12	[K4K15]CZS-1
*Salmonella enterica subsp. enterica serovar* Typhimurium	>512 (>183.17)	512 (184.42)	16 (7.26)

	MBC μg/mL (μM)		
	CZS-9	CZS-12	[K4K15]CZS-1
*Salmonella enterica subsp. enterica serovar* Typhimurium	>512 (>183.17)	>512 (>184.42)	64 (29.04)

**Figure 2:**
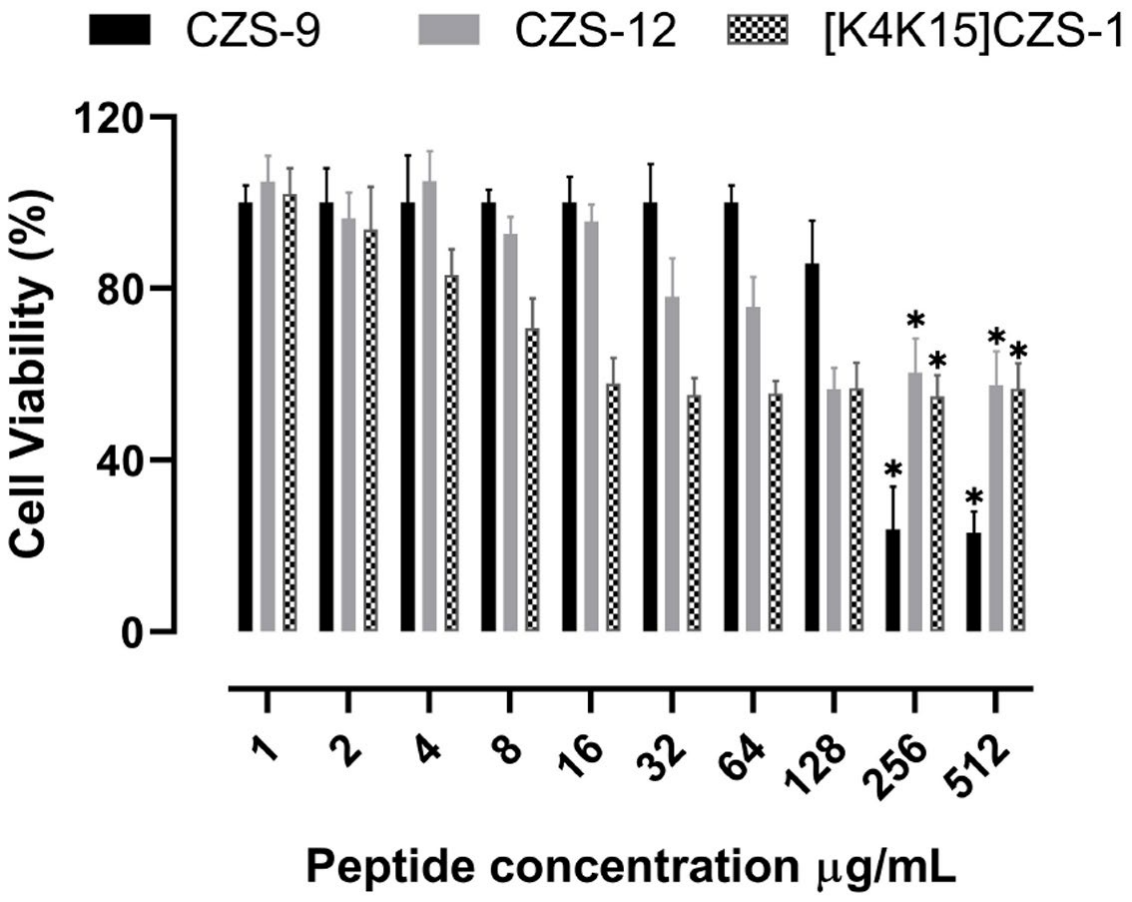
Effect of cruzioseptin family members on cell viability of RAW 264.7 murine macrophage cells. Data are presented as mean ± standard deviation (*n* = 5). Statistically significant difference ANOVA, followed by *post-hoc* Tukey multiple comparisons: *p ≤ 0.05 compared to the untreated control.

The cytotoxicity profiles of these peptides against RAW 264.7 murine macrophage cells were also evaluated after 24 h of incubation using the same concentrations used for MIC assays. The most promising peptide of this study, [K4K15]CZS-1, showed a cytotoxic effect at the MIC concentration (16 μg/mL), with a cell viability percentage of 57.8% (Figure 2). On the other hand, CZS-9 displayed higher toxicity at the highest peptide concentrations (256–512 μg/mL) compared to CZS-12. Given the high antimicrobial activity and moderate toxicity of [K4K15]CZS-1 compared to the other cruzioseptins, this peptide was chosen to perform electron microscopy assays and metabolomics studies to elucidate its full ranges of action against *S.* Typhimurium.

### 3.5 Membranolytic effect of [K4K15]CZS-1

**Figure 3:**
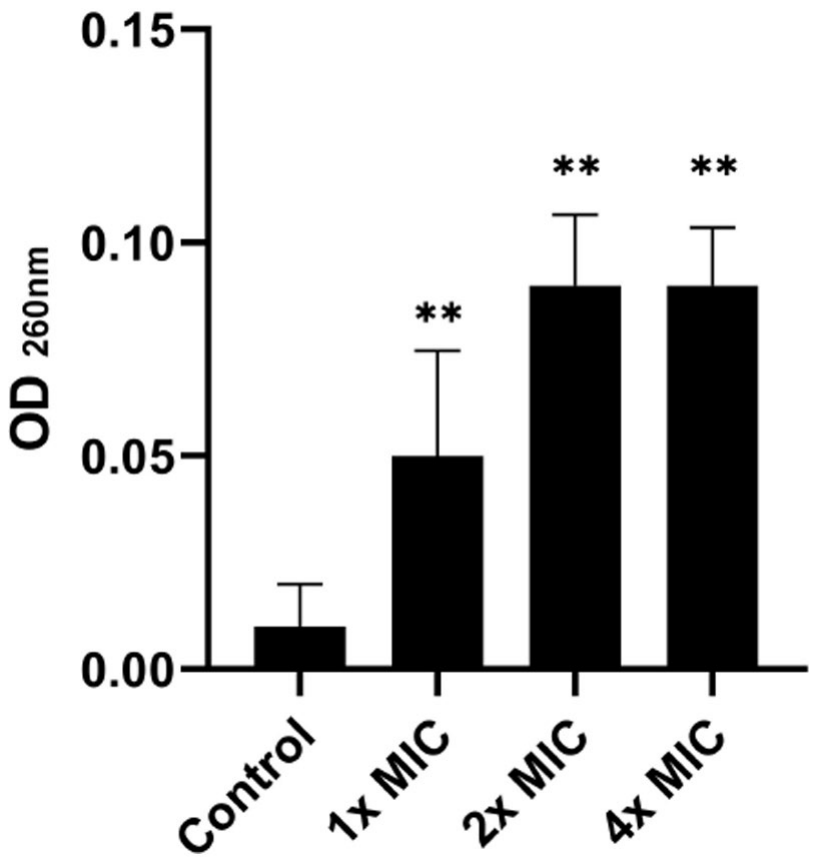
Total nucleotide leakage from *Salmonella enterica subsp. enterica sero*var Typhimurium treated with 1x, 2x, and 4 xMIC of [K4K15]CZS-1. An alteration in the membrane integrity of the pathogen was observed due to the release of nucleic acids. ** Represents comparison with the control group, being ** *p* ≤ 0.01.

The bacterial membrane is the primary target in the mechanism of action of most antimicrobial peptides. In this sense, the release of nucleic acids is considered a good biomarker to evaluate the integrity of the cell membrane, which can be easily monitored through absorbance at 260 nm. As depicted in Figure 3, the nucleic acid leakage assay showed a release of DNA/RNA in a dose-dependent manner. This indicates that the cell membrane of *S.* Typhimurium was compromised by exposure to the [K4K15]CZS-1 treatment.

**Figure 4:**
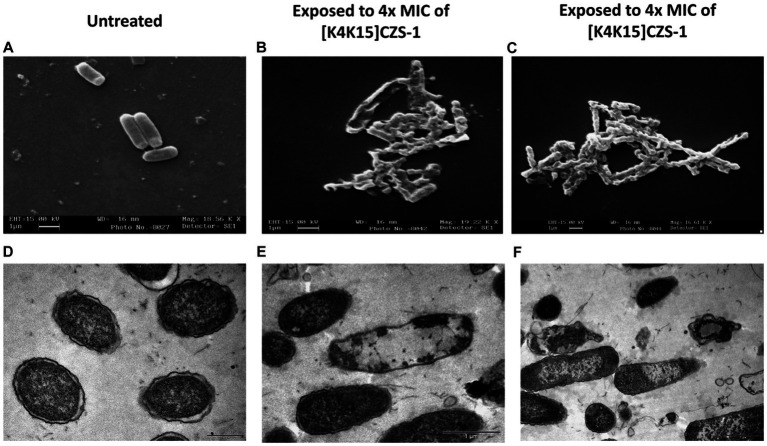
Morphological changes and intracellular alterations in *Salmonella enterica subsp. enterica serovar* Typhimurium exposed to 4 xMIC of [K4K15]CZS-1 was observed by scanning electron microscopy (SEM) and transmission electron microscopy (TEM). SEM micrographs of *S.* Typhimurium: **(A)** no peptide treatment (control) and **(B,C)** [K4K15]CZS-1-treated cells. TEM images of *S.* Typhimurium: **(D)** untreated cells (control), **(E,F)** [K4K15]CZS-1-treated bacterial cells. The scale bar value for **(A–E)** is 1 μm, while for **(D)** it is 0.5 μm.

To corroborate the result above, SEM was performed to visualize the effect of the peptide [K4K15]CZS-1 (4 xMIC) on bacterial membranes. As shown in Figures 4B,C, the morphology of peptide-treated *S.* Typhimurium cells resulted in membrane roughening, corrugation, and cell lysis. On the contrary, a normal shape and smooth surface were observed on the bacterial cells without peptide treatment (Figure 4A).

Likewise, the membrane integrity and alterations in the intracellular content of *Salmonella* before and after peptide treatment were observed using TEM. In Figure 4D, the intact membrane structure and uniform intracellular contents of untreated bacteria are displayed. While *S.* Typhimurium cells after peptide treatment showed a condensed cytoplasmic content and significant changes in the membrane morphology of the bacteria (Figures 4E,F). For instance, TEM images indicated deterioration of cell membranes, cell swelling, and vacuoles composition.

### 3.6 Effect of [K4K15]CZS-1 treatment on intracellular and extracellular metabolite profiles of *Salmonella* Typhimurium

#### 3.6.1 Metabolome analysis

**Figure 5:**
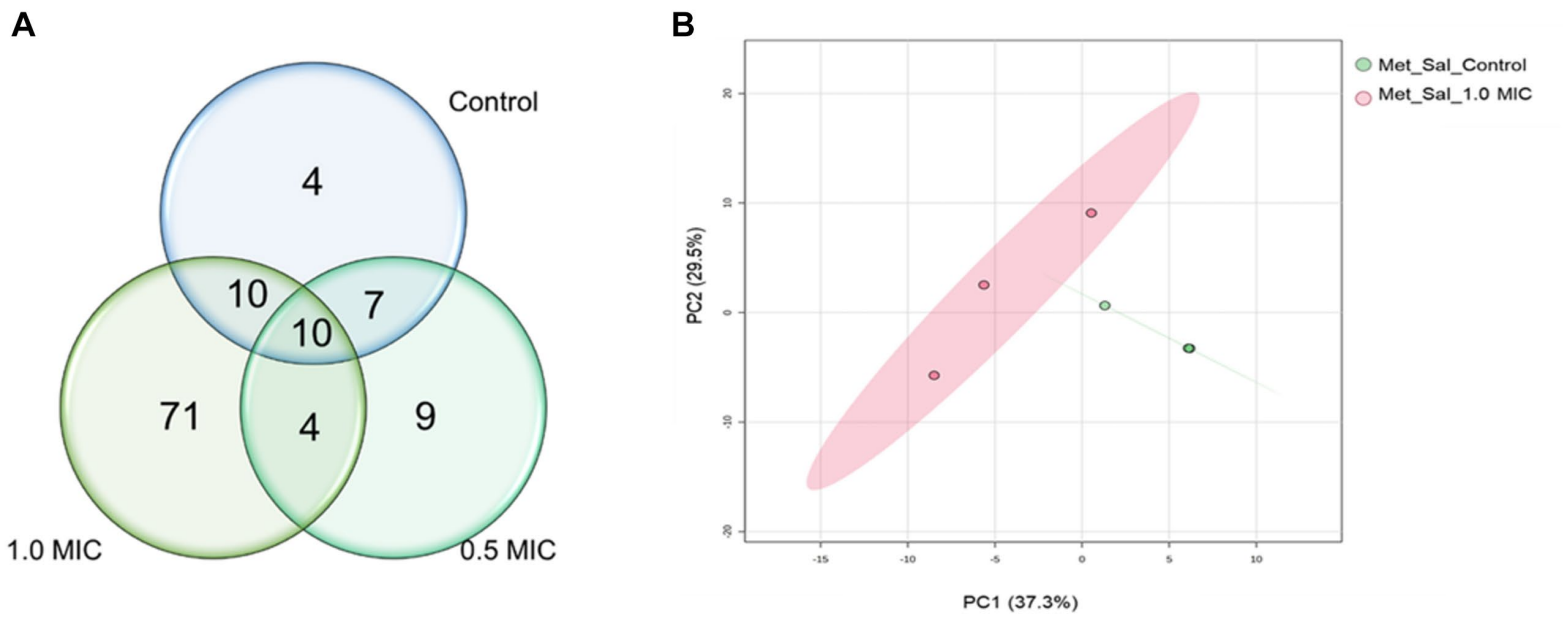
Venn diagram and PCA analysis of intracellular metabolites identified in the samples of *Salmonella enterica subsp. enterica serovar* Typhimurium incubated with [K4K15]CZS-1: **(A)** Venn diagram shows the total of intracellular metabolites of control and [K4K15]CZS-1-treated bacterial cells; and **(B)** the metabolome PCA analysis of *S.* Typhimurium between the control group and treated group with 1.0 xMIC of [K4K15]CZS-1.

The GC–MS results revealed that the quantities of metabolites were higher in *S.* Typhimurium treated with 1.0 xMIC of [K4K15]CZS-1 (Figure 5A). However, when treated with 0.5 xMIC, *S.* Typhimurium showed no differences compared to the control group. The treated group exhibited a remarkable 74.7% difference in metabolites compared to the control group, with metabolite quantities being three times higher than those detected in the untreated *S.* Typhimurium cells. On the other hand, only 20 metabolites were shared between the control and the highest concentration (1 xMIC) of the treatment. The relationship between the control and 1.0 xMIC of [K4K15]CZS-1 treated samples can be observed in the PCA plot depicting PC1/PC2 scores (Figure 5B).

**Figure 6:**
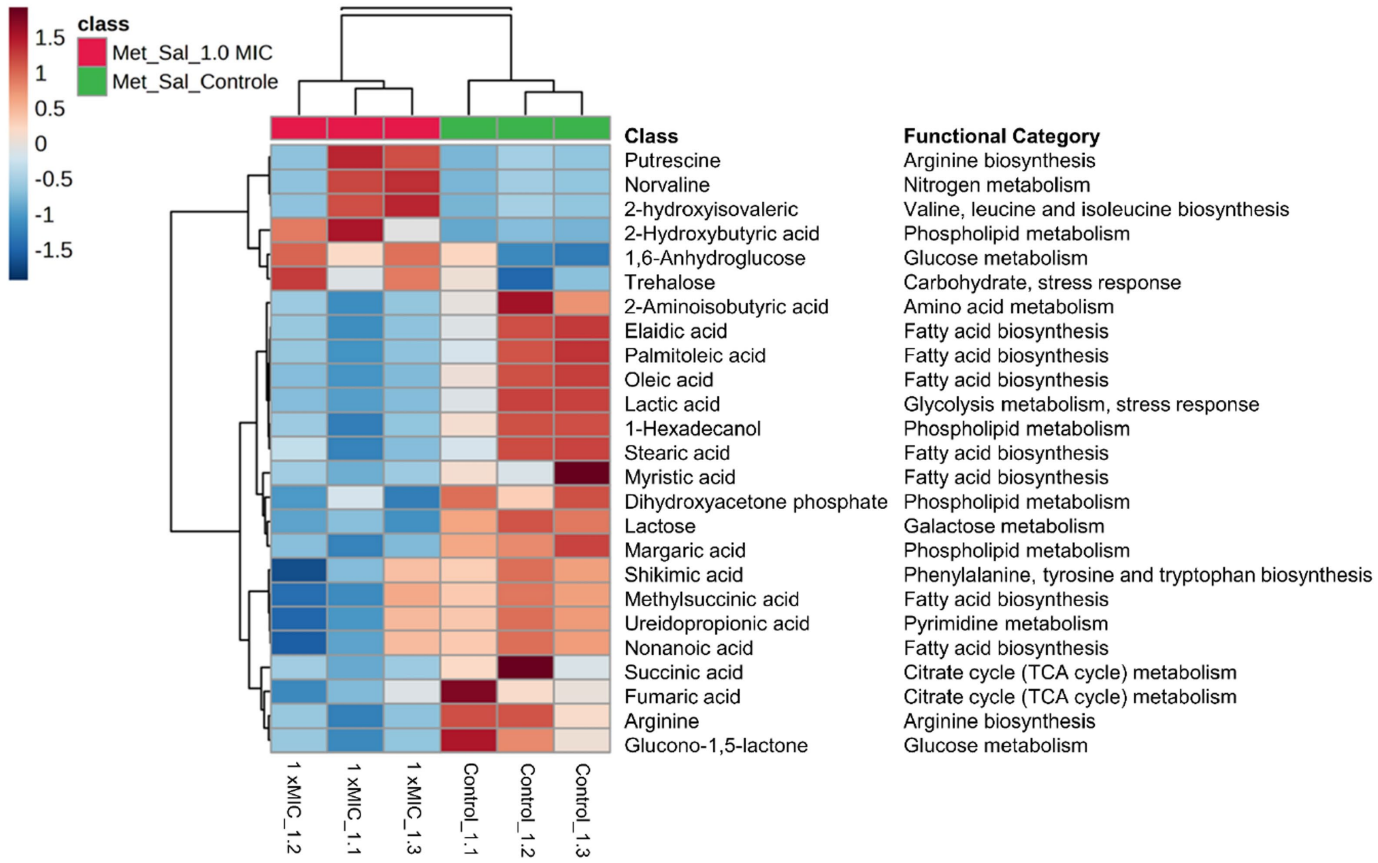
Hierarchical cluster analysis of the 25 most differential metabolites with their respective functional categories. Intracellular metabolites identified in *Salmonella enterica subsp. enterica serovar* Typhimurium treated without or with [K4K15] CZS-1. Red or blue indicate higher or lower relative metabolite abundance, respectively.

For the hierarchical cluster analysis (HCA), a multivariate data analysis was also done through an ANOVA. Based on this, the HCA result is consistent with the PCA analysis, corroborating a similar pattern clustering. In addition, the 25 most significant metabolites were categorized based on their compound type and their position in the metabolic pathway. These categories include amino acids, membrane structure-related compounds, carbohydrates, and alcohols (Figure 6). Among 25 differential metabolites, 6 of them were up-regulated, such as 1,6-Anhydroglucose, 3-Hydroxybutyric acid, 2-Hydroxyisovaleric, norvaline, putrescine, and trehalose, in the treatment with 1 xMIC [K4K15]CZS-1.

The other 19 metabolites were down-regulated, which include several organic acids, fatty acids, carbohydrates, and amino acids. On the other hand, the KEGG enrichment analysis was made to get more information about the metabolic pathway for each differential metabolite (Figure 6). For instance, the up-regulated 1,6-Anhydroglucose and trehalose are related to carbohydrate metabolism. Succinic acid and fumaric acid, two down-regulated metabolites, are intermediates of the tricarboxylic acid (TCA) cycle. While trehalose and lactic acid are associated with cellular stress. The obtained results from the metabolic profiling, which include the identification of significant metabolites with their corresponding fold change trends, were further correlated with the heat map distribution.

#### 3.6.2 Secretome analysis

**Figure 7:**
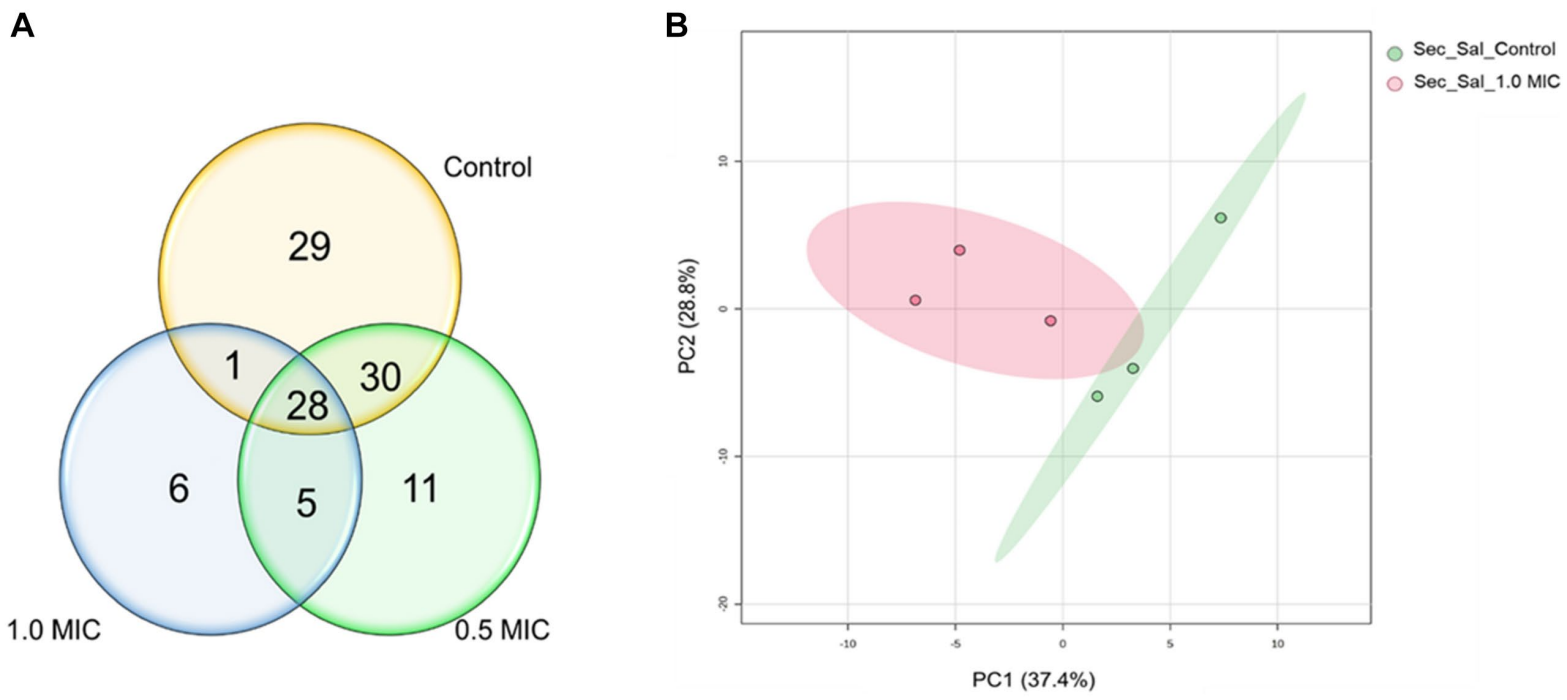
Venn diagram and PCA analysis of the metabolites secreted of *Salmonella enterica subsp. enterica serovar Typhimurium* treated with 0.5 and 1.0 xMIC of [K4K15] CZS-1: **(A)** Venn diagram shows the total of extracellular metabolites of control and peptide treatment and **(B)** PCA score graph of metabolites present in the secretome of *Salmonella enterica subsp. enterica serovar* Typhimurium treated with or without [K4K15]CZS-1 (PC1: 37.4% of total variance; PC2: 28.8% of total variance).

The effect of [K4K15]CZS-1 on the secretome of *S.* Typhimurium was also evaluated through a metabolomic study by GC–MS. Therefore, the metabolites excreted into the extracellular space were analyzed. A total of 88, 74, and 40 metabolites were identified in the control group and in the both peptide treatments (0.5 xMIC and 1 xMIC), respectively (Figure 7A). In this sense, bacteria incubated with AMP suffered a 54.5% reduction in the production of extracellular metabolites.

A multivariate data analysis was performed to visualize the group separation and identify the changes in the extracellular metabolite profile. In fact, there is no significant variation in the metabolite profile induced by 0.5x MIC treatment. Therefore, this data was removed for the subsequent analysis. A PCA analysis of the extracellular metabolites was performed with *S.* Typhimurium in the presence or absence of peptide. The PCA graph demonstrated the clustering of each treatment and clearly shows the separation between them (Figure 7B). In addition, it is possible to observe that the grouping of control samples were the ones that presented a greater influence in PC1, while the samples treated with 1.0 xMIC [K4K15] CZS-1 were the most representative in PC2, since these metabolites were in lower concentrations.

**Figure 8:**
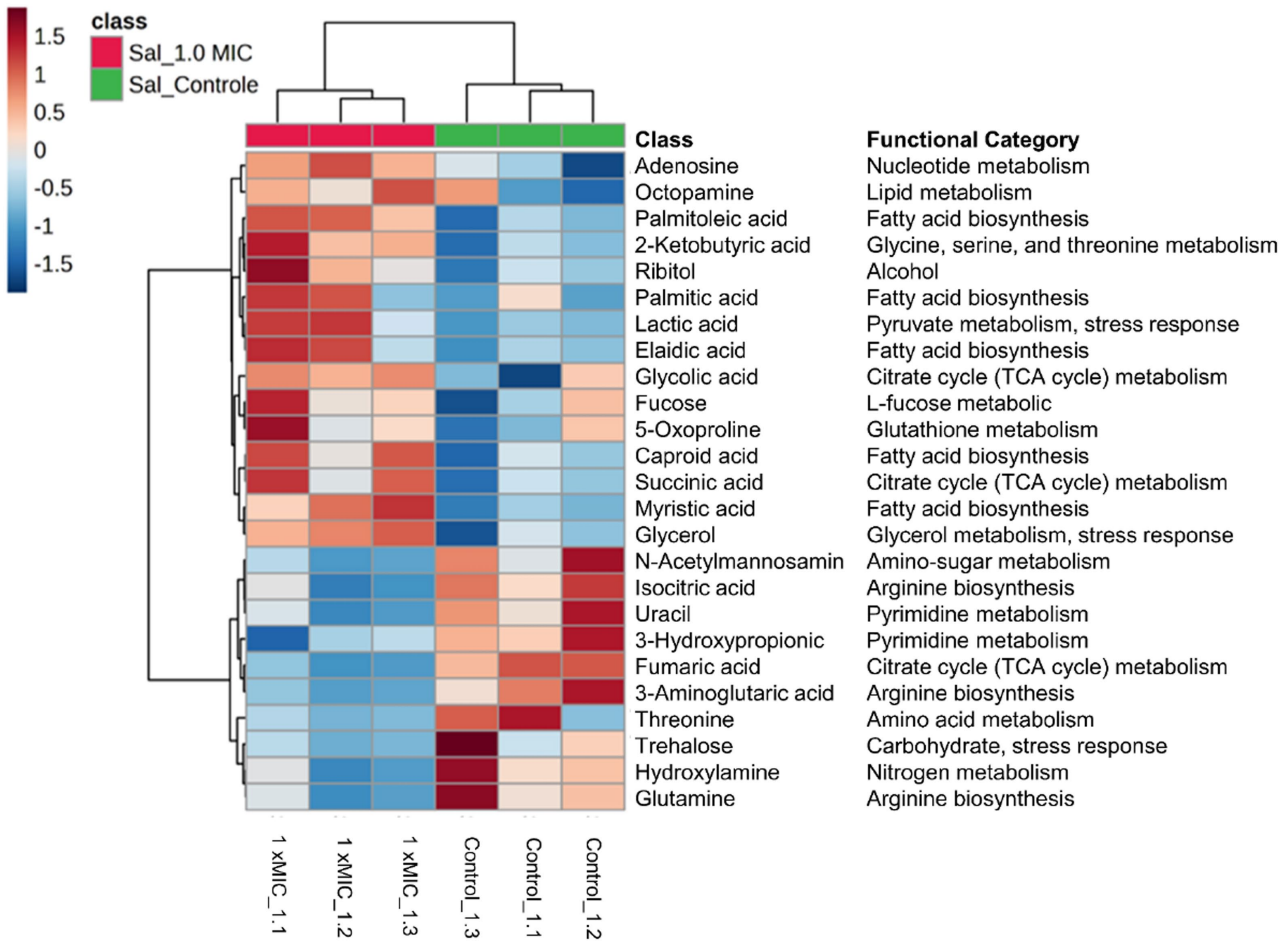
Hierarchical cluster analysis of the 25 most differential metabolites leaking in the secretome of *S.* Typhimurium treated without or with [K4K15] CZS-1. Different colors represent different relative metabolite abundances (red: higher metabolite abundance; blue: lower metabolite abundance).

The HCA showed that there was a significant difference between each group, clustering by treatment, which is a result similar to the PCA analysis. Likewise, the heatmap showed the 25 most significant and different metabolites of the untreated control group and the peptide-treated group (Figure 8). A total of 15 metabolites were more abundant after treatment with 1 xMIC [K4K15]CZS-1, with an increase in the expression of succinic acid, ribitol, palmitoleic acid, palmitic acid, octopamine, N-acetylmannosamine, lactic acid, glycolic acid, glycerol, fucose, elaidic acid, caproic acid, adenosine, 5-oxoproline, and 2-ketobutyric acid. The other 10 metabolites were down-regulated, which include amino acids, carbohydrates, fatty acids, and nitrogenous bases. Subsequently, a KEGG enrichment analysis was conducted to understand the biological pathways involved in the perturbation with the peptide treatment. As shown in Figure 8, several metabolic routes are being up-regulated, such as fatty acid biosynthesis, and lipid metabolism. On the other hand, pyrimidine metabolism and nitrogen metabolism are some pathways that are down-regulated.

## 4 Discussion

In the current study, the anti-Salmonella activity of three cruzioseptins was evaluated. [K4K15]CZS-1 and CZS-12 inhibited the bacterial growth of this pathogen. These findings are consistent with other AMPs (Gong et al., 2010; Festa et al., 2021). For instance, 1,018-K6 inhibited four isolates of *S.* Typhimurium at the same concentration range (16 μg/mL) as [K4K15]CZS-1 (Festa et al., 2021). Intriguingly, CZS-9, did not show antibacterial activity, even at the highest concentration tested (512 μg/mL). Possibly, the difference in the antimicrobial potential between cruzioseptins relies on the positive net charge. CZS-9, CZS-12, and [K4K15]CZS-1 present a net charge of +1, +2, and +6, respectively. In fact, basic amino acids (arginine or lysine) are the main characters involved in the first step of the mechanism of action through an interaction with the negatively charged bacterial membrane (van der Weide et al., 2019; Wang H. et al., 2020). Several authors reported the close relationship between increased cationicity of AMPs and improved antimicrobial activity (Gao et al., 2016; Wang H. et al., 2020; Bermúdez-Puga et al., 2023).

In a translational-biology plan, the assessment of cytotoxicity of peptides in early stages anticipates possible issues in the drug development (Shi et al., 2022). In this context, the toxicity of three cruzioseptins was screened against RAW 264.7 murine macrophage cells using colorimetric cell-based assays. CZS-9 showed reduced cell viability at the two highest tested concentrations compared to CZS-12. Based on the physicochemical parameters of both peptides, CZS-9 is more hydrophobic compared to CZS-12. According to previous studies (Chen et al., 2007; Yang et al., 2013), AMPs with a higher hydrophobicity induced higher toxicity activity against mammalian cells. On the other hand, the cytolytic effect of [K4K15]CZS-1 on RAW 264.7 murine macrophage cells was moderate at the MIC concentration. Several strategies for peptide engineering could be explored to overcome this drawback of [K4K15]CZS-1 (Torres et al., 2018; Kang et al., 2022).

Taking into account the higher antibacterial property of [K4K15]CZS-1, this peptide was chosen to gain insights into the full range of mechanism of action against *S.* Typhimurium. Recently, we demonstrated that [K4K15]CZS-1 acts on the bacterial membrane of Gram-negative *Escherichia coli* (Bermúdez-Puga et al., 2023). Therefore, we initially hypothesized that this molecule also induces membrane damage in *Salmonella*. To corroborate this premise, we combined different experimental strategies, such as a nucleic acid leakage assay and electron microscopy (SEM and TEM). In fact, the results demonstrated that there was a release of DNA and RNA into the extracellular environment, supporting the permeability of the bacterial cell membrane after incubation with this peptide. Likewise, SEM and TEM micrographs exhibited remarkable changes on the bacterial surface compared to untreated cells. This membranolytic effect is in line with the findings of other studies, where permeabilization of the membrane of *Salmonella* spp. is observed after AMP treatment (Lappe et al., 2009; Gong et al., 2010).

TEM observations also revealed that [K4K15]CZS-1 generated intracellular changes in *S.* Typhimurium cells. To create a more comprehensive picture, the metabolic changes were investigated through the analysis of intracellular and extracellular metabolites using an untargeted metabolomic approach by GS-MS combined with multivariate statistical analysis (Supplementary Table S1). The results showed significant differences in the intracellular metabolite profile among the untreated and peptide-treated groups. Bacterial cells incubated with this peptide exhibited a notable reduction in the expression of compounds associated with fatty acid biosynthesis, phospholipid metabolism, and the TCA cycle.

Fatty acids and phospholipids are the major constituents of the bacterial cell wall (Beld et al., 2015; Marquardt et al., 2015), which form a mechanical defense barrier against environmental stress, including antimicrobial compounds (Mueller and Levin, 2020). Here, we observed nine intracellular compounds involved in fatty acid biosynthesis and phospholipid metabolism being down-regulated. This finding shows that [K4K15]CZS-1 might inhibits two important metabolic pathways of the biochemical machinery required to maintain the cell membrane structure. Therefore, this likely impairs the repair of membrane damage induced by synthetic cruzioseptin. In fact, other researchers have also shown that cationic peptides induce variations in the expression of intracellular molecules involved in membrane structure (Bo et al., 2014; Sun et al., 2023). On the other hand, the TCA cycle is a central metabolic pathway which functions as a point of convergence in the catabolism of sugar, lipids, or amino acids in all aerobic microorganisms (Li et al., 2017). [K4K15]CZS-1 negatively affected the TCA cycle, as observed by the low concentration of succinic and fumaric acids. Our findings about the disruption of the energy metabolism are consistent with other studies (Bo et al., 2014; Wang R. et al., 2020).

**Figure 9:**
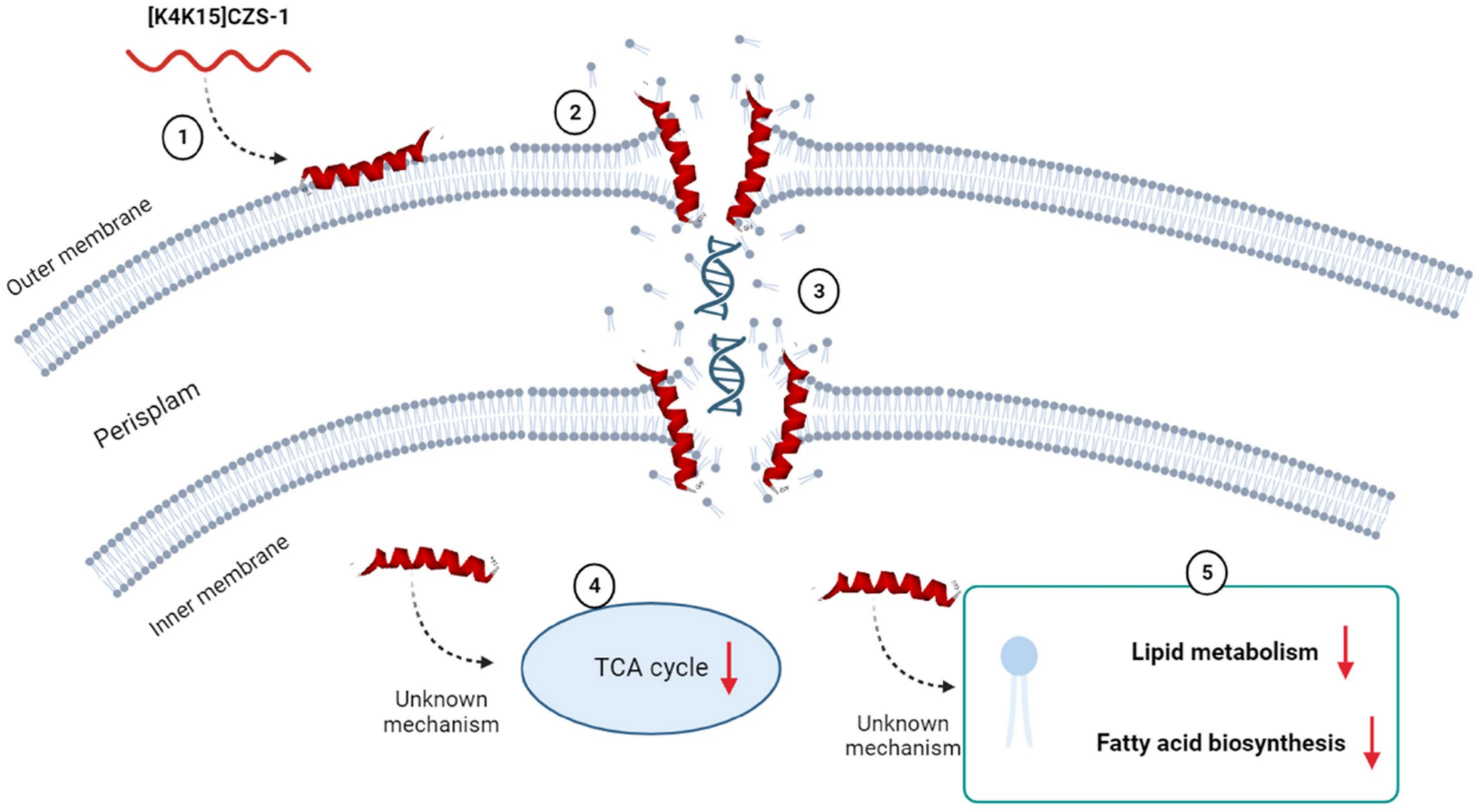
Multidimensional view of the mechanism of action of [K4K15]CZS-1 against *Salmonella enterica subsp. enterica serovar* Typhimurium. The integration of the findings of our multilevel approach is consistent with the proposed model for anti-Salmonella effect. Spectroscopy and microscopy analysis suggest the interaction (1), membrane disruption (2) and subsequent liberation of intracellular content, such as genetic material (3). Mass spectrometry-based approaches reveal the modulation of metabolism pathways (4), including the impairment of membrane repair (5). Image created with Biorender.com.

On the basis of our results, the proposed antibacterial mechanism of [K4K15]CZS-1 against *Salmonella enterica* subsp. *enterica* serovar Typhimurium integrates a series of modulations leading to cell death and minimizing the possibility of resistance. These mechanistic details are schematized in Figure 9. [K4K15]CZS-1 present a random coil conformation in a hydrophilic environment and adopts an α-helix structure after the interaction with membranes (hydrophobic medium). This bacterial membrane-[K4K15]CZS-1 interaction likely occurs through electrostatic attraction between basic amino acids and anionic components of the bacterial membrane. Subsequently, the AMP causes changes in the permeability of the cell membrane and releases the intracellular content, including genetic material. Additionally, [K4K15]CZS-1 acts on intracellular targets, inhibiting the TCA cycle, fatty acid biosynthesis, and phospholipid metabolism. These findings have also been described in earlier investigations. Somes AMPs inhibit the bacterial growth through a dual antibacterial mechanism characterized by membrane-active and intracellular-active interactions (Hsu et al., 2005; Shi et al., 2016).

The study of metabolites presents in the secretome of *S.* Typhimurium also focused on understanding the variations induced by AMP (Supplementary Table S2). This result could help us in exploring the possible mechanism of action and the response generated by the bacteria in the presence of AMP. The extracellular metabolites present in *S.* Typhimurium cells incubated with AMP were significantly different compared to the control group. Metabolic changes, including carbohydrate, alcohol, lipid metabolism, fatty acid biosynthesis, amino acid metabolism, and the TCA cycle, were observed. However, we think that some modifications observed in the composition of the extracellular metabolite profile could be due to bacterial membrane damage and subsequently the release of intracellular content. For instance, a high expression of several molecules that are synthesized inside the cell was visualized, such as adenosine, glycolic and succinic acids.

Metabolomics analysis also revealed the presence of various protective compounds (Supplementary Table S2). For instance, trehalose is a non-glucose reducing disaccharide, generally considered a protective substance of the cell membrane. It can also function as an antioxidant, an osmoregulatory solute, or a carbohydrate reserve that can be mobilized during times of stress (Hounsa et al., 1998; Benaroudj et al., 2001; Ye et al., 2012). Glycerol is another compound with a protective function. Glycerol acts as a modulator, which can help to transfer compounds across the cell wall. On the other hand, studies have shown that several microorganisms cope with the toxic effects of reactive oxygen species (ROS) by accumulating non-reducing disaccharides such as trehalose in the intracellular medium as well as polyalcohols such as glycerol and arabitol (Sánchez-Fresneda et al., 2013; Galdiero et al., 2021). It is known that in organisms grown in an aerobic environment, the formation of ROS is a consequence of an active oxidative metabolism or the presence of external hazardous agents (Cabiscol et al., 2000). Therefore, the presence of both compounds in the peptide-treated group may indicate a metabolic response generated due to the stress.

## 5 Conclusion

[K4K15]CZS-1 exhibited promising antibacterial properties, which can benefit the development of innovative strategies to prevent *Salmonella*-induced food poisoning. A multidimensional view of the mechanism of action of bioactive cruzioseptin through complementary approaches evidenced a broad effect integrating membranolysis, modulation of metabolic pathways, and intracellular targeting activities. This expands the current landscape of mechanism of action of antimicrobial cruzioseptins and reinforces the multiple strategies used by AMPs to avoid bacteria resistance. Taking these findings together, they motivate further investigations focused on the stability and structure–function-guided optimizations of cruzioseptins to enrich the probability of success through the biotechnological application of peptide-based agents on food industry.

## Data availability statement

The original contributions presented in the study are included in the article/Supplementary files, further inquiries can be directed to the corresponding author.

## Author contributions

SB-P: Conceptualization, Formal analysis, Investigation, Methodology, Visualization, Writing – original draft. MD: Investigation, Methodology, Writing – original draft. TF: Investigation, Methodology, Writing – original draft. CM: Investigation, Writing – original draft. SY: Investigation, Writing – original draft. ER: Investigation, Writing – review & editing. CN: Investigation, Methodology, Writing – review & editing. MM: Investigation, Methodology, Writing – review & editing. PO: Writing – review & editing. JA: Formal analysis, Writing – review & editing. CP-B: Formal analysis, Investigation, Writing – review & editing. RO: Conceptualization, Formal analysis, Funding acquisition, Investigation, Project administration, Supervision, Writing – review & editing.

## Footnotes

^1^Retrieved from: https://www.bachem.com/knowledge-center/peptide-calculator/, 5 July 2023.
