# Supplementary material for: Dual antibacterial mechanism of [K4K15]CZS-1 against Salmonella Typhimurium: a membrane active and intracellular-targeting antimicrobial peptide
Source: Front Microbiol. 2023 Dec 14;14:1320154. doi: 10.3389/fmicb.2023.1320154 (PMC10752938; doi:10.3389/fmicb.2023.1320154)
Supplement: Supplementary file 3 [file Data_Sheet_1.docx]

**Supplementary Material**


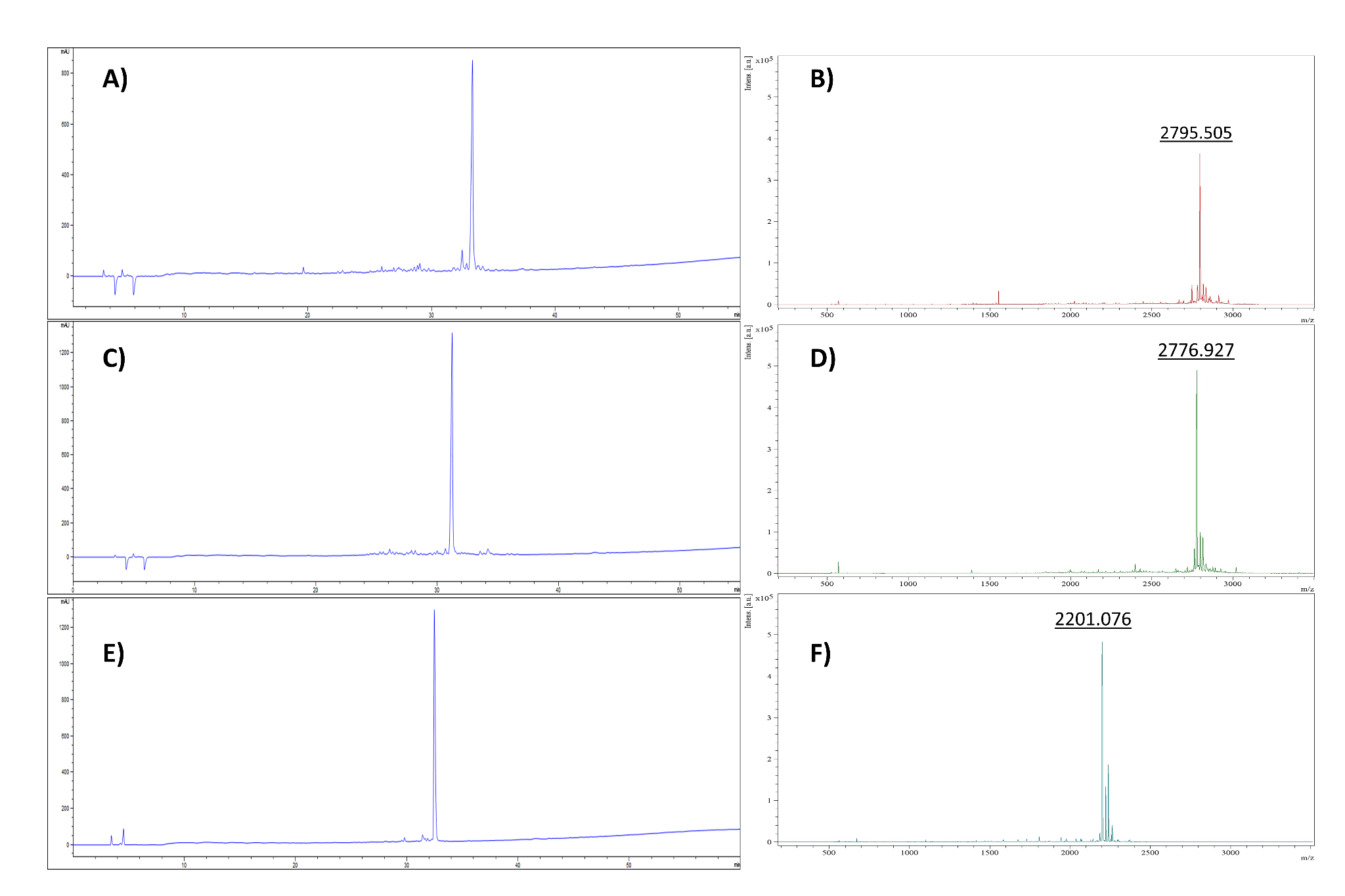


**Supplementary Figure 1.** Chromatographic profiles and MALDI-TOF MS spectra of the three synthetic peptides: **(A - B)** CZS-9; **(C-D)** CZS-12; and **(E-F)** [K4K15]CZS-1
